# Changes in Abrasion Resistance of Cast Cr-Ni Steel as a Result of the Formation of Niobium Carbides in Alloy Matrix

**DOI:** 10.3390/ma16041726

**Published:** 2023-02-19

**Authors:** Grzegorz Tęcza

**Affiliations:** Department of Cast Alloys and Composites Engineering, Faculty of Foundry Engineering, AGH University of Science and Technology, 30 Mickiewicza Ave., 30-059 Krakow, Poland; tecza@agh.edu.pl

**Keywords:** cast chromium-nickel steel, microstructure, primary carbides, niobium carbides, heat treatment, hardness, abrasive wear

## Abstract

Cast austenitic chromium-nickel steel is commonly used for the manufacture of machine parts and components, which are exposed to the attack of corrosive media and abrasive wear during operation. The most commonly used grades include GX2CrNi18-9 and X10CrNi18-8 as well as GX2CrNiMo17-12-2 and X6CrNiMoNb17-12-2. To improve the abrasion resistance of cast chromium-nickel steel, primary niobium carbides were produced in the metallurgical process by increasing the carbon content and adding Fe-Nb. The microstructure of the obtained test castings consisted of an austenitic matrix and primary niobium carbides evenly distributed in this matrix. The measured hardness of the samples after heat treatment ranged from 215 to 240 HV and was higher by about 60 units than the hardness of the reference cast GX10CrNi18-9 steel, which had a hardness of about 180 HV. Compared to the reference cast steel, the abrasive wear resistance of the tested cast chromium-nickel steel (measured in Miller test) with contents of 4.4 and 5.4 wt% Nb increased only slightly, i.e., by 5% for the lower niobium content and 11% for the higher niobium content. Compared to ordinary cast GX10CrNi18-9 steel, the addition of 9.2 wt% Nb reduced the abrasive wear by almost 2.5 times.

## 1. Introduction

Austenitic steels and cast chromium-nickel steels are used for the manufacture of machine parts and components operating under the conditions of corrosive attack and abrasive wear. The most commonly used grades include GX2CrNi18-9 and X10CrNi18-8 as well as GX2CrNiMo17-12-2 and X6CrNiMoNb17-12-2, all of them being designed for service in numerous branches of the industry. These materials are used not only in the chemical, cellulose, and paper industries or in food processing plants, but also in heavy industry, including mining and materials processing sectors, where they operate as large-size castings often exposed to the effect of abrasion in devices such as heat exchangers, tanks, feeders, screws, and transmission pipelines. Castings made of these materials also operate in pumps and distributors for pumping out suspensions of water and sand, sludge, and also brine. Additionally, when operating as components of the drilling platform equipment, valves, tees, or clamps, they are sometimes exposed to the effect of low temperature combined with impact stresses [1,2,3,4].

Table 1 gives examples of the chemical composition of austenitic and austenitic-ferritic corrosion-resistant steels. The chemical composition of cast steel grades corresponds to the chemical composition of steel.

The addition of strong carbide-forming elements such as Ti or Nb to these alloys is at the level of tenths of a percent (maximum up to 1%) and is dictated by the technological process. For example, 0.2% Ti acts as a modifier and 0.5% Nb is added to improve technological and functional properties such as weldability or corrosion resistance.

The microstructure of the cast steels presented in Table 1 should be austenitic (or austenitic-ferritic) and free from chromium carbide precipitates at the grain boundaries, as their presence adversely affects the casting resistance to intergranular corrosion. This type of structure is obtained by the application of heat treatment, namely solution treatment of the alloy.

**Figure 1:**
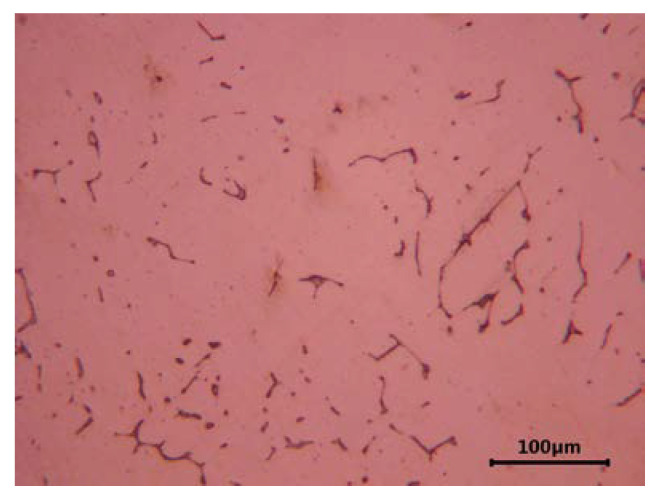
Microstructure of cast GX10CrNi18-9 steel after solution treatment; austenite with traces of ferrite; bright color—austenite, dark color—ferrite, etched with Mi16Fe [author].

**Figure 2:**
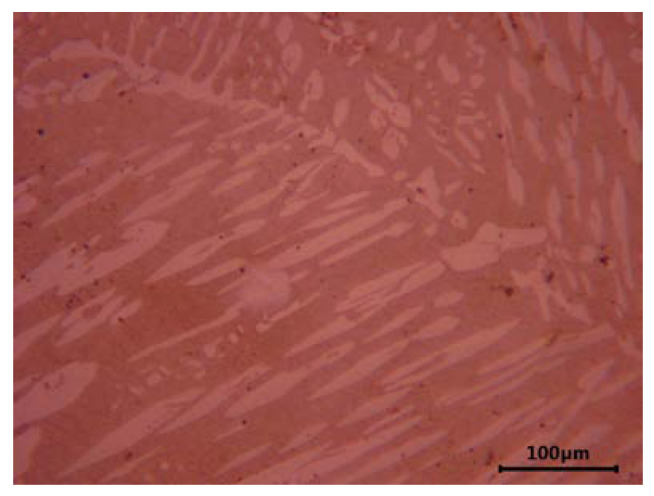
Microstructure of cast GX6CrNiMo19-10-3 steel after solution treatment; cast duplex steel, bright color—austenite, dark color—ferrite, etched with Mi16Fe [author].

Unlike steels that are usually austenitic, corrosion-resistant cast steels with the same chemical composition contain small amounts of delta ferrite in their microstructure. Its presence results from the slow cooling of castings and segregation processes occurring during their solidification. In castings made of the austenitic Cr-Ni steel, 5–25% of evenly distributed delta ferrite is often found in the alloy matrix. The amount of this constituent depends on the thickness of the casting wall. The presence of delta ferrite is often desirable owing to its beneficial effect on the weldability of castings and the ability to hinder the spread of microcracks and improve the resistance to intergranular and stress corrosion (it favors the precipitation of carbides inside its own grains and not at the ferrite-austenite interface) [4]. In alloys with the addition of molybdenum, the volume fraction of delta ferrite is even higher and distinctly visible. Figure 1 and Figure 2 show the characteristic microstructure of ferrite-containing cast austenitic steel after solution treatment. Different amounts of delta ferrite are observed in the cast steel without and with the addition of molybdenum (Figure 1 and Figure 2, respectively).

**Figure 3:**
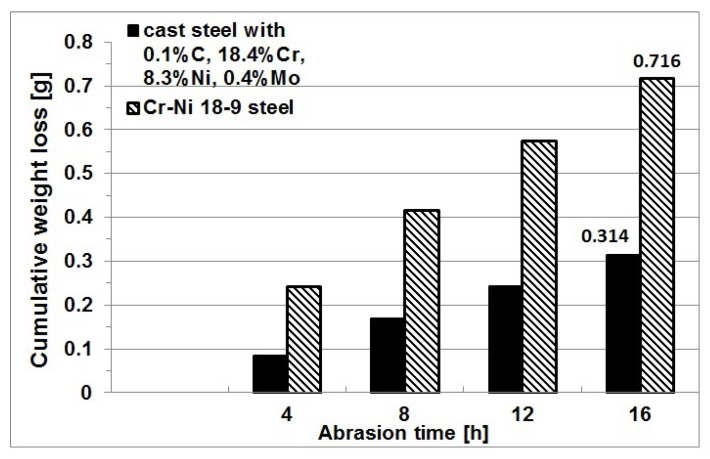
Weight losses as a function of abrasion time in Miller test compared for samples of Cr-Ni 18-9 steel and cast steel [author].

Similar results of microscopic examinations were obtained by the authors in studies [6,7,8,9,10]. For example, in study [6], in addition to the characteristics of the microstructure of selected cast Cr-Ni steel grades, the results of hardness and impact strength measurements at −40 °C and +20 °C were presented. On the other hand, [7] gave the results of the modification of cast austenitic Cr-Ni 18-9 steel with mischmetal (Rare Earth Metals) in the ladle and its impact on the microstructure. The tests were carried out on industrial melts in which the delta ferrite structure was obtained with the carbon content of 0.1%. The author’s own research on the wear behavior of various Cr-Ni alloys showed another very important characteristic that gives castings a strong competitive advantage. The point is that castings operating under the conditions of sand abrasion offer a wear resistance superior to that of steel (even twofold increase). Figure 3 compares the wear resistance of heat-treated cast austenitic Cr-Ni steel containing 0.1% C, 18.4% Cr, 8.3% Ni, and 0.4% Mo with the wear resistance of steel characterized by a similar chemical composition.

A review of the literature discussing problems of the wear behavior of various materials indicates that current research and development activities mainly focus on the study of dual-phase and multi-phase alloys, including cast austenitic steel whose matrix is reinforced with carbides and nitrides [11,12,13,14]. More and more often, precision castings for use as parts of industrial machines exposed to rapid wear are expected to keep their dimensions within narrow tolerances; otherwise, the efficiency of the entire device decreases and parts must be replaced with new ones. Frequent downtimes and replacement of large-size castings generate high costs, so new materials are sought that would meet the highest tribological requirements. In the currently conducted research, a tendency evolves to keep the cast alloy matrix ductile, while hardening only the surface of the casting. These design solutions can be achieved in various ways, e.g., by the well-known method of explosive hardening of the surface of high-manganese steel castings [15], or making castings with abrasion-resistant composite zones by the SHS powder synthesis (Self-propagating High-temperature Synthesis). In the latter case, in selected zones of the casting, carbides are formed in liquid alloy as a result of the reaction proceeding under the effect of high temperature created in the alloy and carbides synthesis from the mixture of powders [16,17].

In [18], the results of experimental studies are discussed, showing that changes in the microstructure of cast austenitic 18%Cr-9%Ni steel as a result of the addition of about 1.4% boron and 1.4% boron with titanium increase the wear resistance of this steel grade. It was demonstrated that the matrix of cast austenitic steel contained titanium nitride precipitates and a eutectic rich in boron and chromium with a microhardness close to 1900 HV0.02. The changes that took place in the microstructure increased the hardness of cast steel matrix from 212 HV to 350 HV. In the 16 h Miller test (ASTM G75-07), which is used to compare the wear resistance of different materials, an over 20% increase in wear resistance was achieved.

The search for new techniques and technologies of tool production prompted the author to develop a concept that would meet the assumption that tool steels and cast steels may have satisfactory wear resistance, ductility, and crack resistance even at high or low temperatures. These properties are due to the presence of MC and M2C carbides that occur in an austenitic matrix of the alloy. An evident drawback is the presence of “coarse” (thick) MC carbides in castings, significantly deteriorating the cast steel ductility [19,20,21].

Following the example of tool steels, the essence of the solution proposed by the author consists in the use of a technology that allows the particles of primary carbides to be formed during metallurgical process within the entire volume of steel melt and later in the casting. In earlier research [21,22,23,24] on the properties of cast austenitic high-manganese steel with the addition of vanadium, titanium, and niobium, the author and co-workers focused on changes in the microstructure and abrasion resistance (determined in Miller test) of cast high-manganese steel. The structure obtained in the tested cases after heat treatment consisted of an austenitic or austenitic-martensitic matrix with primary carbides of the introduced elements evenly distributed in this matrix. The abrasion resistance measured in the Miller test was at least twice as high as in the reference cast Hadfield steel.

The idea of introducing carbide-forming elements, successfully applied in cast high-manganese steel, and the satisfactory results of changes in microstructure and abrasion resistance obtained in the Miller test prompted the author to conduct similar research on cast chromium-nickel steel with the addition of titanium [25], where, in test castings, the microstructure consisting of an austenitic Cr-Ni matrix with evenly distributed titanium carbides was obtained. After casting and solution treatment, the hardness ranged from 300 to 330 HV0.02 and was higher by about 40–70 units than the hardness of the reference cast GX2CrNi18-9 steel, which amounted to 258 HV0.02. The wear resistance of the tested cast Cr-Ni steel measured in the Miller test increased by at least 20% with the content of 1.3 wt% Ti. Compared to ordinary cast GX2CrNi18-9 steel, increasing the titanium content to 5.3 wt% and 6.9 wt% reduced the wear by 2.5 times.

The main topic discussed in the present study is the effect of niobium addition to cast Cr-Ni steel on the above-mentioned properties of the obtained alloys.

## 2. Materials and Methods

**Figure 4:**
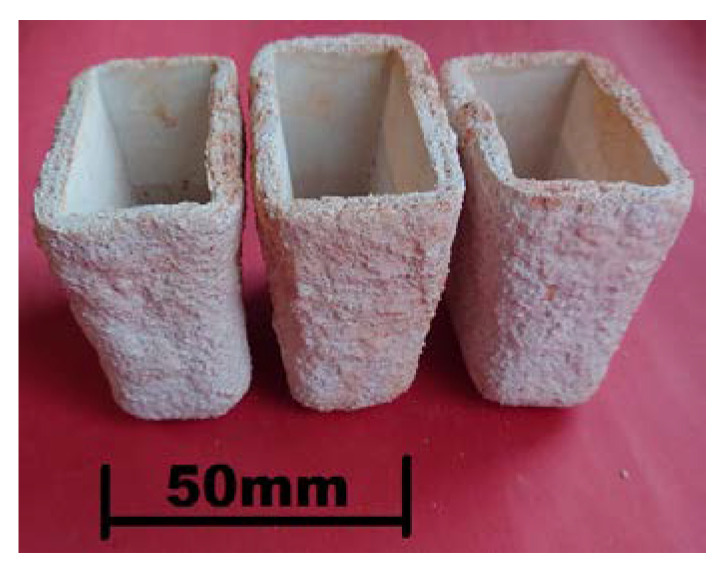
Ceramic molds used in tests.

**Figure 5:**
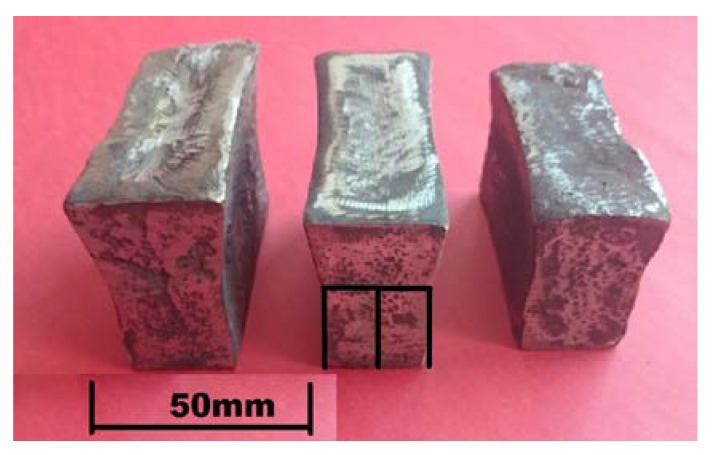
“Y”-type test castings with marked places where specimens were cut out for tests.

Test melts were carried out in a Balzers VSG-02 laboratory induction vacuum furnace (Balzers, Bergisch Gladbach, Germany) using a 1 kg capacity Al_2_O_3_ crucible. The charge was 18-10 steel scrap and high-purity pig iron of known chemical composition used as a carburizer. After melting the charge, to obtain the required chemical composition, alloy additives such as the Fe-Mn65 and Fe-Si65 ferroalloys, metallic chromium, and electrolytic nickel were successively introduced. After melting the alloy additives, stirring the melt, and heating to 1600 °C, the metal was deoxidized with aluminum added in the amount of 1 g/1 kg of steel [26]. In the final stage of melting, Fe-Nb60 ground to the size of 3–5 mm was added in portions to avoid the temperature drop in molten steel. Niobium addition to the steel melt triggered the formation of primary niobium carbides. After casting solidification, these carbides were evenly distributed in the alloy matrix. Thus, processed melt was held in the furnace for 6 min, and shortly before pouring of molds, it was deoxidized again with Fe-Ca-Si added in the amount of 1 g/1 kg of steel. The pouring temperature was 1560–1570 °C and the steel was poured into ceramic molds made by the lost-wax process. The inner layer was made of quartz flour and the outer layer of quartz sand. Ethyl silicate and Ludox AM-30 silica gel were used alternately as a binder. The ceramic molds were fired in a chamber kiln at 950 °C to create bonds in the mold material and increase the strength. Before pouring, the molds were heated to 200–250 °C. Figure 4 shows the ceramic molds ready for pouring with molten alloys, while Figure 5 shows the “Y”-type test castings with a wall thickness of 25 mm, length of 70 mm, and a weight of about 0.8–0.9 kg. A diagram of cutting out the specimens for subsequent heat treatment, microstructure examinations, and wear tests is also included.

Chemical analysis of the tested alloys was carried out under industrial conditions using a Spectro Maxx LMF04 spectrometer (Spectro, Kieve, Germany) and was next completed with laboratory examinations using a Spectro Midex energy-dispersive X-ray fluorescence spectrometer (Spectro, Kieve, Germany). Table 2 shows the chemical composition of the tested alloys.

Chemical analysis of the composition of test samples showed that the content of the main elements, i.e., Mn, Cr, and Ni, in the melted cast steel was comparable to the content of these elements in the reference cast GX10CrNiMo18-9 steel. High silicon content (from 0.6 to 0.9%) was the result of double deoxidation with Fe-Ca-Si and aluminum, where the content of the latter one at the level of 0.03–0.04% indicates correct deoxidation of the steel melt. The content of niobium in the three tested specimens was 4.4%, 5.4%, and 9.2% and increased with the increasing carbon content, which was 0.3%, 0.5%, and 0.8%, respectively. The Nb/C ratio was, in each case, above 10, which guarantees complete bonding of carbon into carbides and the absence of cementite in the microstructure of the tested alloys. The specimens cut out for testing were subjected to solution treatment, i.e., water cooling from the temperature of 1050 °C and holding for 30 min.

Hardness was measured with a Vickers hardness tester (Werkstoffpruefmaschinen, Leipzig, Germany) under a standard load of 30 kg, applied to both as-cast samples and samples after solution treatment.

The microstructure of the tested alloys was examined under a Neophot 32 light microscope (Carl Zeiss Jena, Hövelhof, Germany) equipped with a camera for digital image recording. Chemical analysis of the composition of carbides present in the tested alloys and of the alloy matrix was performed with a JEOL JSM 7100 F secondary electron (SE) field emission scanning electron microscope (SEM) made in Tokyo, Japan, and with a scanning electron microscope equipped with an EDS detector supplied by Oxford Instruments in Abingdon, UK.

Phases present in the tested samples were identified with a Kristalloflex 4H X-ray diffractometer from Siemens, Munich, Germany, using the characteristic Cu radiation (Kα = 0.154 nm) with a step size of 0.052 theta/1 s.

**Figure 6:**
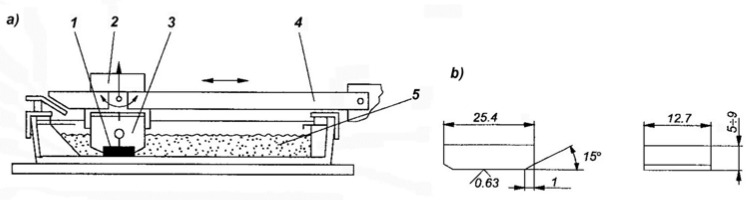
Diagram of the machine used in the Miller test. (**a**) 1: specimen, 2: weight, 3: specimen holder, 4: holder arm, and 5: abrasive. (**b**) Dimensions of the specimen tested for abrasive wear.

The abrasive wear response was determined in the Miller test conforming to ASTM G75, which is used to compare the abrasive wear behavior of various construction materials. Figure 6 shows a diagram of the Miller tester.

The applied method of wear testing allows the author to compare the currently obtained results with the results of their own experiments conducted previously, and with the results obtained by other research teams [18,21,22,24,25,26,27,28,29,30,31]; however, the latter is possible only when the tests are conducted under identical conditions of abrasion. In these studies, in most cases, the conditions of the conducted experiments were not precisely specified, and therefore, the author could compare the obtained results only with their previous research.

The test samples with dimensions of 25.4 × 12.7 mm and a thickness of 9 mm were placed in the holders of the device under a constant load of 22.2 N and were next subjected to abrasion in a mixture of water and silicon carbide in a 1:1 ratio. The counter-sample was the rubber lining of the trough bottom where the abrasion process took place. Silicon carbide with grain number 220 according to the FEPA standard and a grain size of 53–73 µm was used. Two 16 h abrasion tests were performed in 4 cycles for each sample, calculating next the mean. Every four hours, the samples were weighed with an accuracy of 0.001 g. Based on the obtained values of weight losses, abrasive wear curves were plotted for the tested samples. The values of wear obtained for the samples of the tested alloys were compared with the values of wear obtained for the reference sample cast from the GX10CrNi18-9 steel containing 0.1% C, 18.4% Cr, 8.3% Ni, and 0.4% Mo, subjected to standard heat treatment, i.e., solution treatment in water from the temperature of 1050 °C, and characterized by the hardness of 180 HV.

The surfaces of the samples after abrasive wear tests were macroscopically compared with the surface of the reference sample cast from the GX10CrNi18-9 steel.

Detailed analyses of the wear mechanism operating in the tested cast steel and of the surface condition of samples after the abrasion test require additional profilometric tests, which is a standard method used for the assessment of surface conditions after various technological and tribological operations. Examinations of the surface condition after wear test will be the subject of further studies carried out by the author.

## 3. Test Results and Discussion

In this study, various issues related to the microstructure characterization, hardness, and wear resistance of cast austenitic Cr-Ni steels with primary niobium carbides produced in the metallurgical process are discussed.

**Figure 7:**
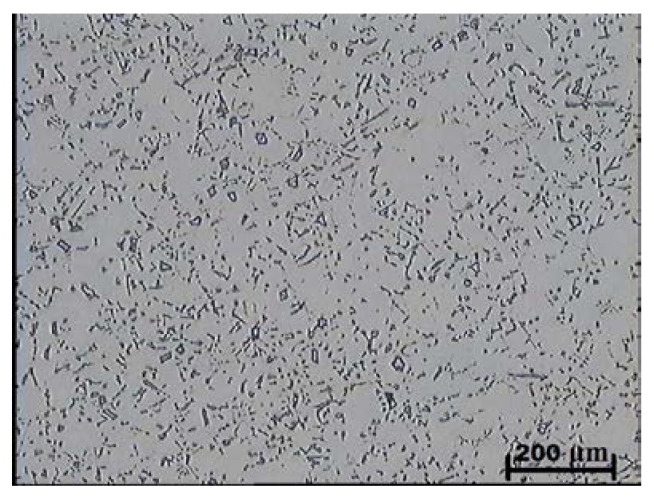
As-cast microstructure obtained in the alloy with 5.4% Nb addition; austenitic matrix with primary niobium carbides; etched with Mi16Fe.

**Figure 8:**
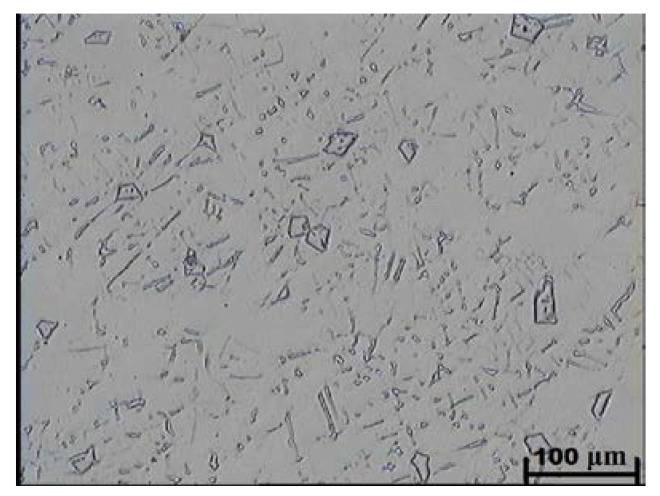
As-cast microstructure obtained in the alloy with 9.2% Nb addition; austenitic matrix with primary niobium carbides; etched with Mi16Fe.

Examinations using light microscopy showed that the as-cast microstructure of the tested alloys with the niobium content of 4.4–9.2% consisted of an austenitic matrix and plate-like shaped primary niobium carbides evenly distributed in this matrix. The presence of cementite was not traced in the examined microsections. Figure 7 and Figure 8 show examples of as-cast microstructures obtained in the test castings containing 5.4 and 9.2% Nb. The as-cast hardness of the tested alloys amounted to about 210 HV and was independent of the carbon content and niobium addition (Table 3).

The tested samples were subjected to a heat treatment (solution treatment in water), and then their hardness was measured. The heat treatment parameters and the measured hardness values are given in Table 3.

**Figure 9:**
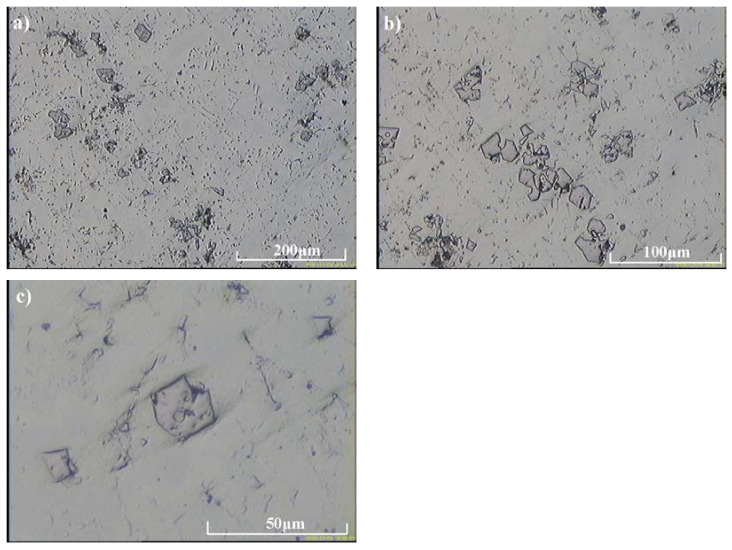
Microstructure obtained in the alloy with 9.2% Nb addition after solution treatment; austenitic matrix with niobium carbides: (**a**) 400×, (**b**) 800×, (**c**) 2000×, etched with Mi16Fe.

**Figure 10:**
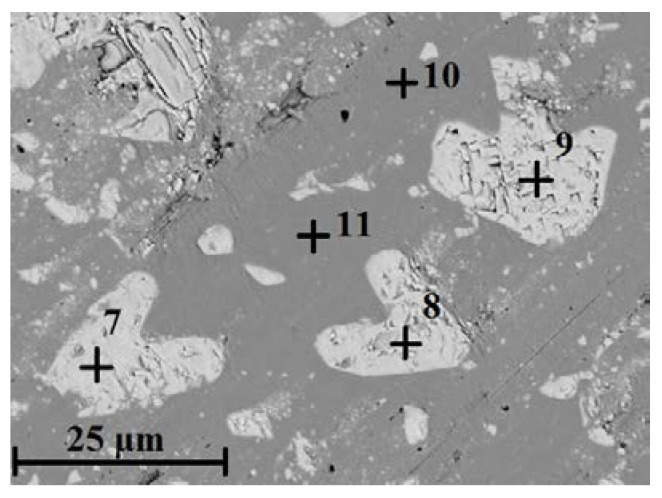
Sample scanning image in the alloy with 9.2% Nb addition after solution treatment; austenitic matrix with niobium carbides, etched with Mi16Fe.

**Figure 11:**
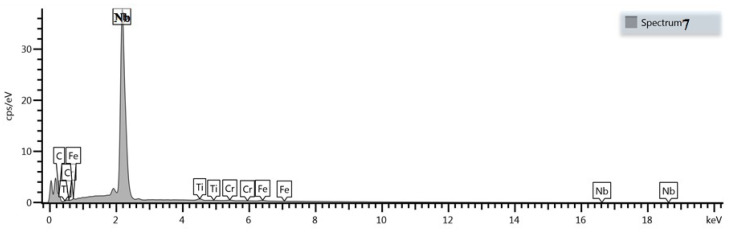
Sample EDS spectrum of niobium carbide precipitates obtained for the alloy containing 9.2% Nb shown in Figure 10.

**Figure 12:**
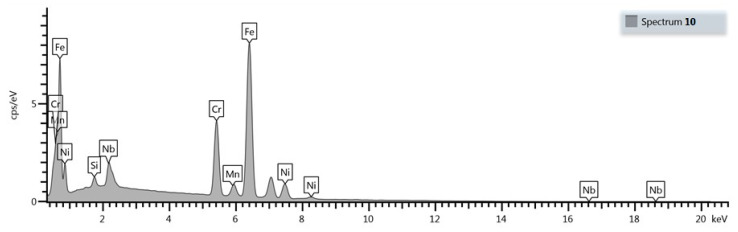
Sample EDS spectrum of the alloy matrix containing 9.2% Nb shown in Figure 10.

**Figure 13:**
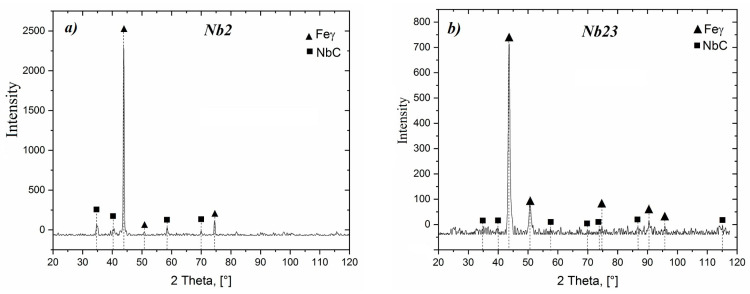
X-ray diffraction patterns of alloys containing: 9.2% Nb—(**a**) and 4.4% Nb—(**b**).

**Figure 14:**
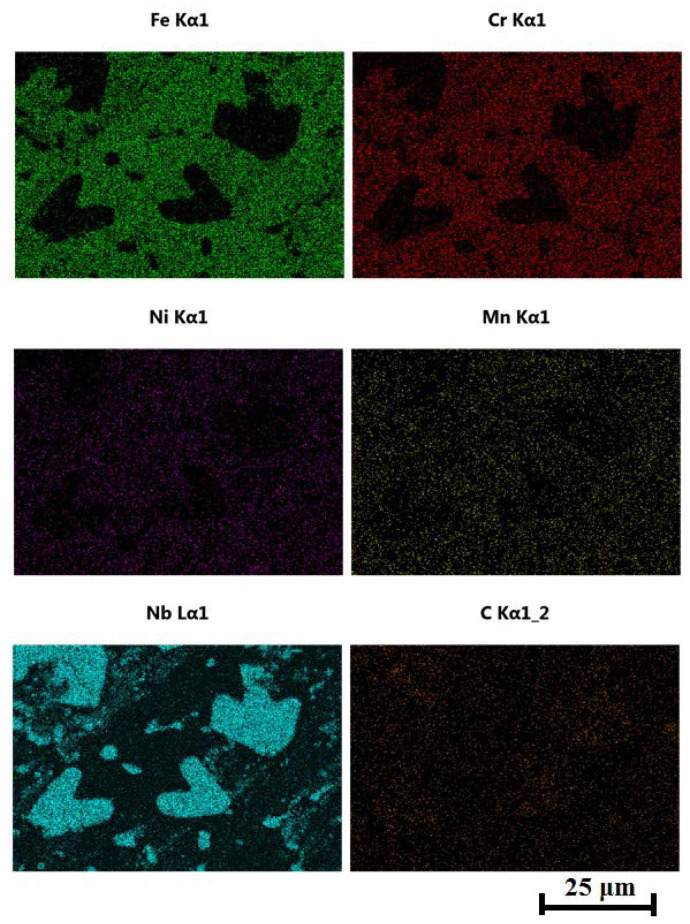
Surface distribution of elements such as Fe, Cr, Ni, Mn, Nb, and C in the area of niobium carbides present in the alloy with 9.2% Nb content shown in Figure 10.

Compared with as-cast samples, the solution treatment from the temperature of 1050 °C followed by cooling in water caused an increase in the obtained values of hardness, which further increased with the increasing content of carbon and niobium. In samples with the lowest content of carbon and niobium, the hardness after solution treatment increased by about 5 HV units, while for the niobium content of 5.4%, the increase in hardness amounted to about 15 HV units. The highest hardness was obtained in samples with the niobium content of 9.2%. Its value exceeded by 30 HV units the hardness obtained in as-cast state. The small scatter of the measured hardness values proves the homogeneity of the tested alloys and even distribution of carbide precipitates in the alloy matrix. This is also the reason why the average hardness values are not given in Table 3. The results of combined studies, including examinations by light microscopy at various magnifications (Figure 9) and scanning microscopy (Figure 10), chemical analysis of carbides (Figure 11, Table 4) and the alloy matrix (Figure 12, Table 4), and X-ray phase analysis (Figure 13) and the surface distribution of elements such as Fe, Cr, Ni, Mn, Nb, and C in the area of the visible carbides (Figure 14), demonstrated that the microstructure of all tested cast steels after solution treatment consisted of an austenitic matrix and primary niobium carbides evenly distributed in this matrix. Precipitates of cementite were not traced in the tested samples. Carbides in the areas of primary grain boundaries showed the tendency to form small clusters (Figure 9a,b).

### Wear Resistance

**Figure 15:**
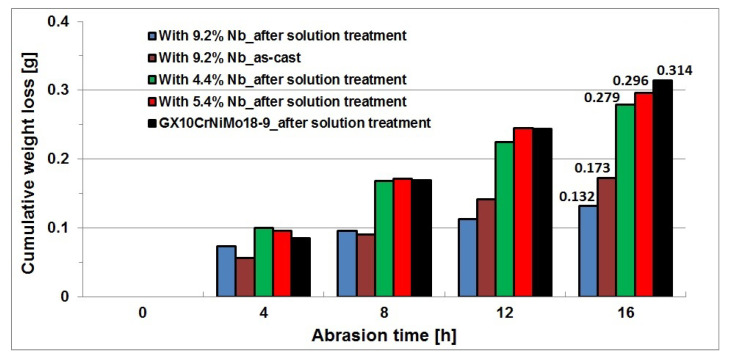
Cumulative weight loss of samples of the tested alloys as a function of abrasion time.

The abrasion resistance was measured in a Miller test on samples after solution treatment from the temperature of 1050 °C, as this type of heat treatment is recommended as a standard procedure for cast Cr-Ni steel, and on the sample with the highest niobium content, i.e., 9.2%, in the as-cast state. From the obtained partial weight losses of samples, the total weight loss was calculated for each test cycle, and using these data, a graph was plotted to show the cumulative weight loss of samples as a function of abrasion time. Figure 15 shows the cumulative weight loss of test samples plotted against the abrasion time.

From the obtained results, it follows that the wear resistance of the tested cast chromium-nickel steel increased with the increasing content of niobium addition, but for the contents of 4.4 and 5.4%, this increase was insignificant. The value of wear decreased from 0.314 g/16 h for the reference cast steel to 0.296 g/16 h for the content of 5.4% Nb and to 0.279 g/16 h for the content of 4.4% Nb. Compared to the reference cast GX10CrNiMo18-9 steel, the increase in niobium content to 9.2% reduced the wear almost twice (even in as-cast state) and then its value amounted to 0.173 g/16 h. The lowest wear of 0.132 g/16 h was obtained for samples of this alloy after solution treatment in water.

**Figure 16:**
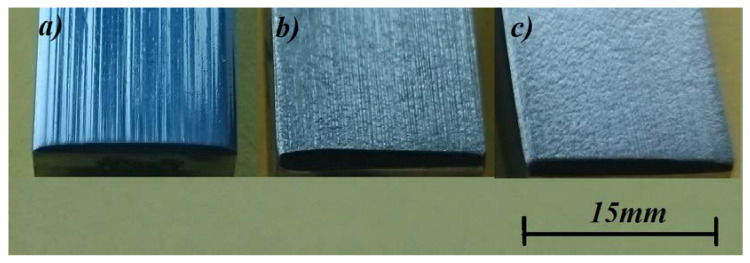
Surface of samples after the abrasion process; (**a**)—reference sample of cast GX10CrNiMo18-9 steel, (**b**)—cast Cr-Ni steel with the addition of 5.4% Nb, (**c**)—cast Cr-Ni steel with the addition of 9.2% Nb.

Figure 16 shows the macroscopic images of the surfaces of samples after the abrasion test compared with the reference sample made of cast chromium-nickel steel. In the cast GX10CrNiMo18-9 steel, deep scratches and grooves were visible on the sample surface, which still preserved its glossy and smooth appearance (Figure 16a). This grade of cast steel with a purely austenitic structure (similar to cast high-manganese steel) underwent the abrasion wear process by furrowing. The addition of niobium in an amount of at least 4.4% made the wear of the samples more uniform—no cracks or furrows appeared and the surface of the samples was even and slightly rough (Figure 16b,c), which proves that the wear process in the tested samples took place through matrix losses. Further analysis of the wear mechanism and surface condition requires additional profilometric tests, which will be conducted as part of the next research program.

## 4. Conclusions

The results of the studies discussed in this article lead to the conclusion that the introduction of strongly carbide-forming elements, such as niobium, into the molten chromium-nickel steel produced primary carbides in the alloy matrix, the presence of which increased, sometimes even many times, the wear resistance of the steel. When properly satisfied, this condition allows the tested alloy to be used for new material applications, mainly the tools and components exposed to wear. As shown by the results of microstructural examinations, in the majority of cases, the obtained niobium carbides were of a faceted type. They were also characterized by a slight tendency to form clusters in the interdendritic spaces. This distribution of carbides in the steel matrix may increase the risk of crack formation in the resultant castings and reduce their impact strength. Therefore, the next stage of the research on particle-reinforced cast steel should be devoted to the development of a method of the steel melting and modification that will allow the changing of the size, shape, and distribution of primary carbides in the matrix. The promising results obtained so far allow the following conclusions to be drawn:

1. Primary niobium carbides produced in molten steel were evenly distributed in the austenitic matrix, but in the interdendritic spaces, their tendency to form small clusters was observed.
2. No precipitation of cementite was observed in the tested samples after heat treatment.
3. The measured as-cast hardness of the tested samples amounted to about 210 HV and was independent of both carbon content and niobium addition.
4. After heat treatment, the hardness of the tested samples increased with the increase in niobium content, ranging from 215 to 240 HV.
5. The formation of primary niobium carbides in the matrix of chromium-nickel cast steel increased the abrasion resistance by even 2.5 times.
6. Carbides evenly distributed in the austenitic chromium-nickel matrix changed the wear behavior of the tested samples. Owing to their presence, the surfaces of the produced samples were free from any furrows and deep scratches.

## Figures and Tables

**Table 1:** Chemical composition of austenitic chromium-nickel steels [5].

Alloy Designation	Chemical Composition (wt%)									
	C	Mn	Si	P	S	Cr	Ni	Mo	Fe	Other
X2CrNi18-9	<0.03	<2.00	<0.80	<0.045	<0.030	18.5	8.8	–	Bal.	N < 0.11
X5CrNi18-10	<0.07	<2.00	<0.80	<0.045	<0.030	18.5	9.3	–	Bal.	N < 0.11
X10CrNi18-8	<0.10	<2.00	<1.00	<0.045	<0.030	17.5	7.8	<0.8	Bal.	N < 0.11
X6CrNiTi18-10	<0.08	<2.00	<0.80	<0.045	<0.030	18.0	10.5	–	Bal.	Ti = 5 × C − 0.7
X2CrNiMo17-12-2	<0.03	<2.00	<1.00	<0.045	<0.030	17.5	11.5	2.3	Bal.	N < 0.11
X6CrNiMoTi17-12-2	<0.08	<2.00	<1.00	<0.045	<0.030	17.5	12.0	2.3	Bal.	Nb = 10 × C − 1.0

**Table 2:** Chemical composition of the tested cast Cr-Ni-Nb steel.

Alloy Designation	Chemical Composition (wt%)										
	C	Mn	Si	P	S	Cr	Ni	Mo	Al	Nb	Fe
Nb2	0.8	1.3	0.9	0.04	0.01	16.8	11.6	1.5	0.03	9.2	Bal.
Nb22	0.5	1.3	0.7	0.04	0.02	17.6	10.3	1.3	0.04	5.4	Bal.
Nb23	0.3	1.8	0.6	0.03	0.01	18.0	8.6	0.3	0.03	4.4	Bal.

**Table 3:** Heat treatment of the tested alloys and respective hardness values.

Alloy Designation	Heat Treatment	Hardness (HV)
Nb2	As-cast	210, 212, 213, 212
	1050 °C/0.5 h/water	241, 241, 237, 239
Nb22	As-cast	213, 210, 213, 212
	1050 °C/0.7 h/water	227, 218, 234, 226
Nb23	As-cast	213, 204, 201, 210
	1050 °C/0.5 h/water	216, 214, 220, 216

**Table 4:** Chemical composition in the areas examined at points 7–11 in Figure 10.

Place of Analysis	(wt%)								
	C	Si	Ti	Cr	Mn	Fe	Ni	Nb	Total
Point 7	8.2	–	1.1	0.6	–	1.1	–	89.0	100.0
Point 8	10.1	–	1.0	0.5	–	1.0	–	87.4	100.0
Point 9	10.3	–	1.2	0.5	–	0.9	–	87.1	100.0
Point 10	–	0.7	–	17.6	0.9	66.8	10.5	3.5	100.0
Point 11	–	0.7	–	18.1	1.1	68.1	10.8	1.2	100.0

## Data Availability

Data sharing not applicable, all the data created for this study are already displayed in the article.
